# Wnt signaling, de novo lipogenesis, adipogenesis and ectopic fat

**DOI:** 10.18632/oncotarget.2769

**Published:** 2014-11-15

**Authors:** Kangxing Song, Shuxia Wang, Mitra Mani, Arya Mani

**Affiliations:** ^1^ Cardiovascular Research Center, Yale University School of Medicine, New Haven, CT; ^2^ Cornell University, Ithaca, NY; ^3^ Department of Genetics, Yale University School of Medicine, New Haven, CT

## Abstract

Wnt signaling is as a major regulator of adipogenesis. It differentially regulates the fate of mesenchymal stem cells (MSC) by promoting osteogenesis and myogenesis, and inhibiting adipogenesis[1]. Its loss of function has been associated with impaired osteogenesis[2] and diverse congenital and adult cardiovascular disorders[3,4]. Our group has identified loss of function mutations in Wnt coreceptor LRP6 that underlie autosomal dominant early onset coronary artery (CAD), osteoporosis and most features of the metabolic syndrome, including high plasma triglyceride and LDL-C, diabetes, hypertension, hyperuricemia and fatty liver disease (unpublished data). Following we will describe our most pertinent findings related to Wnt/LRP6 regulation of de novo lipogenesis and adipogenesis and the role of impaired Wnt signaling in generation of ectopic fat, insulin resistance, elevated plasma lipids and non-alcoholic fatty liver disease (NAFLD).

## Wnt and LRP6 regulation of insulin receptor and insulin signaling

LRP6^R611C^ mutation carriers exhibit impaired total body insulin sensitivity compared to noncarrier relatives in response to oral glucose ingestion, which correlates with a significant decline in their skeletal muscle expression of the insulin receptor (IR). Further investigations showed that this mutation diminishes TCF7L2-dependent transcription of the IR, whereas it increases the stability of IGFR and enhances mTORC1 pathway activity[5]. Overexpression of TCF7L2 in patient's skin fibroblasts was able to rescue the IR expression. The study was not sufficiently powered to assess hepatic glucose output and was not designed to examine the role of LRP6 in regulation of IR in pancreas or liver. Others have shown the important role of LRP6 in increasing the expression of IR and decreasing the expression of IGF1R in preadipocytes[6]. Interestingly, Ras/Src-transformed tumors in a drosophila model of diet induced hyperglycemia and insulin resistance retained insulin sensitivity and displayed aggressive behavior toward increased metastases by increasing insulin receptor expression and preferential glucose uptake[7]. Wnt activation in Ras/Src-transformed tumors upregulated the insulin receptor gene expression by promoting the expression of the transcript factor TCF7L2. This mechanism may provide one explanation for higher prevalence of malignant tumors in patients with diabetes and/or obesity. Paradoxically, mice with global TCF7L2 overexpression exhibit insulin resistance[8]. One explanation is that in this model excess TCF7L2 protein is unbound to β-catenin and acts as a transcription suppressor.

On the other hand, activation of the noncanonical Wnt results in increased PKC activity, which can lead to decreased insulin receptor kinase activity and insulin-stimulated IRS tyrosine phosphorylation and subsequent reduced insulin sensitivity. This ultimately leads to decreased insulin-stimulated hepatic glucose uptake and reduced insulin suppressibility of hepatic glucose production.

### Wnt and de novo lipogenesis

**Figure 1:**
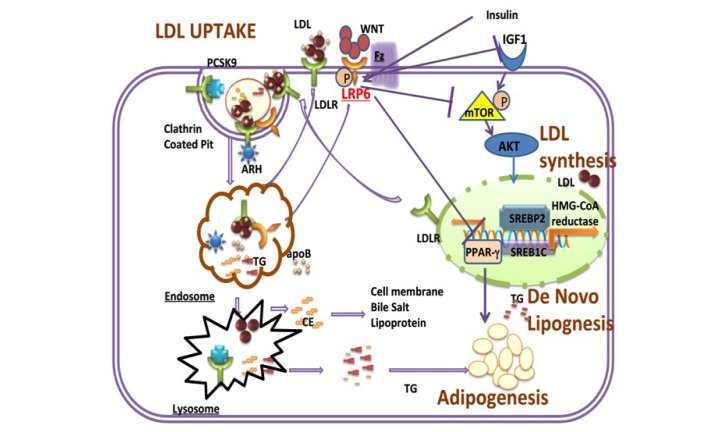
Schematic of LRP6 Regulation of LDL uptake and synthesis, de novo lipogenesis and adipogenesis LRP6 forms a complex with LDLR and other proteins of endocytic machinery involved in LDL uptake. It also inhibits IGF1-IGF1R dependent activation of AKT/SREBP pathway and inhibits DNL and PPARγ-dependent adipogenesis. DNL; De novo lipogenesis.

Studies in mice with LRP6^R611C^ mutation have shown that this mutation triggers hepatic de novo lipogenesis, lipid and cholesterol biosynthesis, and ApoB secretion by Sp1-dependent activation of IGF1, AKT, and both mTORC1 and mTORC2 axis (Figure 1). Strikingly, these pathways could be normalized after treatment of primary hepatocytes with Wnt3a. Administration of rmWnt3a to LRP6^R611C^ mice was able to normalize plasma TG and LDL levels[9]. Mice with LRP6^R611C^ mutation also developed steatosis and insulin resistance of the liver, which could were similarly rescued with Wnt3a treatment (same reference).

### Wnt, adipogenesis and lipotoxicity

Excessive caloric intake results in enhanced adipogenesis via adipocyte hypertrophy and hyperplasia. When excess energy outweighs the capacity of the adipose tissue to accumulate fat, triglycerides spill over to ectopic tissues and organs and leads to lipotoxicity and organ damage. Ectopic fat is defined as excess TG storage in tissues other than adipose tissue, such as the liver, skeletal muscle, heart, and pancreas. With the advent of magnetic resonance spectroscopy (MRS) and noninvasive quantification of tissue fat, excess intramyocellular, intrahepatocellular, myocardial and pancreatic lipids have has been discovered in subjects with insulin resistance. The extent of ectopic fat and triglyceride content of the liver and skeletal muscle tightly correlates with plasma lipid profile and whole-body and tissue-specific insulin sensitivity and its excess has been associated with diabetes, CAD, cerebrovascular disease and sudden death risk. Whether ectopic fat is the cause or the result of insulin resistance is unknown and hence, its pathogenesis is poorly understood[10].

**Figure 2:**
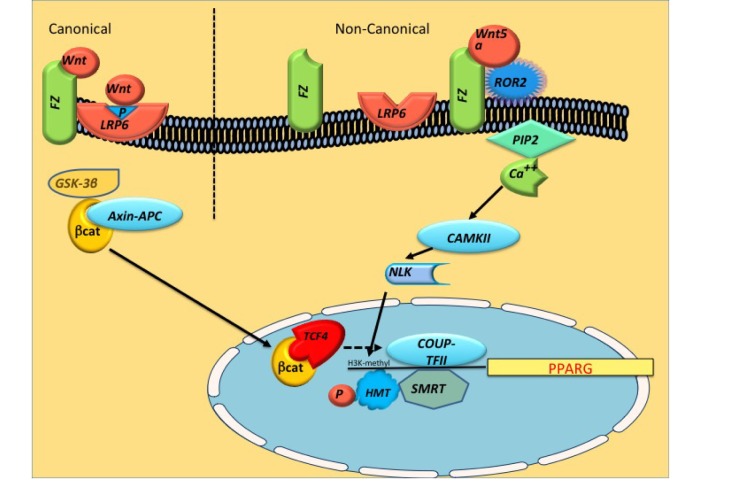
Schematic of canonical and noncanonical Wnt pathways leading to deacetylation of PPARgamma (PPARG) Noncanonical Wnt-5a activates NLK, which phosphorylates a HMT, resulting in inhibition of PPARG transcription through histone H3-K9 methylation The canonical Wnt/beta-catenin signaling enhances the expression of COUP-TFII, which recruits the SMRT to the first intron of both PPARgamma1 and gamma2 gene, reducing their acetylation and repressing their expression. HMT (histone methyl transferase), SMRT (silencing mediator of retinoic acid and thyroid hormone receptor), NLK (Nemo-Like Kinase), COUP-TFII (COUP transcription factor 2), SETDB1 (SET domain bifurcated 1).

Adipocyte hyperplasia is grossly divided into two stages of mesenchymal stem cells (MSCs) “commitment” to preadipocytes and preadipocytes differentiation to adipocytes. Mesenchymal stem cell fate is regulated by a network of extracellular signaling factors that determine the activity of lineage-specific transcription factors. Both canonical and noncanonical Wnt inhibit adipogenesis in both stages (Figure 2) through deacetylation of PPARγ and C/EBPα promoter and blocking of their expressions[1]. MRS and ultrasound studies and tissue biopsies in LRP6 mutation have revealed excess intramyocellular an intrahepatic fat (data not published). Interestingly, recent anthropomorphic examinations have revealed that LRP6 mutation carriers have visceral obesity despite normal BMI. Accordingly, mice with LRP6^R611C^ mutation exhibit ectopic fat in the liver[9], kidney and heart (data not published), whereas heterozygote LRP6 knockout mice are protected against diet-induced obesity and are insulin sensitive[11]. TG storage within the cardiomyocytes is known as myocardial steatosis and can be measured with great sensitivity by ^1^H-MRS[12]. Patients with IGT and T2DM have an increased myocardial TG content compared to obese and lean controls, which may explain increased incidence of noni-ischemic heart disease and diastolic dysfunction in these subjects[13]. Similarly, increased fat in kidney has been reported in subjects with insulin resistance and is considered to be a cause of hypertension.

### Problems and Prospects

LRP6 is ubiquitously expressed and has pleiotropic effects. Most studies on Wnt signaling pathway and its role in glucose homeostasis, adipogenesis and energy expenditure have been conducted in animal models in a tissue specific manner. Additional studies in humans are required to validate the findings in animal models and to resolve the controversies surrounding them. On the other hand, development of novel therapeutics for lipotoxicity and insulin resistance with no or minimal systemic effects requires future identification of tissue specific targets within Wnt pathway.

## References

[1] Bowers R, Lane M (2008). Wnt signaling and adipocyte lineage commitment. Cell Cycle.

[2] Gong Y, Slee RB, Fukai N, Rawadi G, Roman-Roman S (2001). LDL receptor-related protein 5 (LRP5) affects bone accrual and eye development. Cell.

[3] Mani A, Radhakrishnan J, Wang H, Mani M, Nelson-Williams C (2007). LRP6 mutation in a family with early coronary disease and metabolic risk factors. Science.

[4] Singh R, Smith E, Fathzadeh M, Liu W, Go GW (2013). Rare nonconservative LRP6 mutations are associated with metabolic syndrome. Hum Mutat.

[5] Singh R, De Aguiar RB, Naik S, Mani S, Ostadsharif K (2013). LRP6 Enhances Glucose Metabolism by Promoting TCF7L2-Dependent Insulin Receptor Expression and IGF Receptor Stabilization in Humans. Cell Metab.

[6] Palsgaard J, Emanuelli B, Winnay JN, Sumara G, Karsenty G (2012). Cross-talk between insulin and Wnt signaling in preadipocytes: role of Wnt co-receptor low density lipoprotein receptor-related protein-5 (LRP5). J Biol Chem.

[7] Hirabayashi S, Baranski TJ, Cagan RL (2013). Transformed Drosophila Cells Evade Diet-Mediated Insulin Resistance through Wingless Signaling. Cell.

[8] Savic D, Ye H, Aneas I, Park SY, Bell GI (2011). Alterations in TCF7L2 expression define its role as a key regulator of glucose metabolism. Genome Res.

[9] Go GW, Srivastava R, Hernandez-Ono A, Gang G, Smith SB (2014). The Combined Hyperlipidemia Caused by Impaired Wnt-LRP6 Signaling Is Reversed by Wnt3a Rescue. Cell Metab.

[10] Shimabukuro M, Kozuka C, Taira S, Yabiku K, Dagvasumberel M (2013). Ectopic fat deposition and global cardiometabolic risk: new paradigm in cardiovascular medicine. J Med Invest.

[11] Liu W, Singh R, Choi CS, Lee HY, Keramati AR (2012). Low density lipoprotein (LDL) receptor-related protein 6 (LRP6) regulates body fat and glucose homeostasis by modulating nutrient sensing pathways and mitochondrial energy expenditure. J Biol Chem.

[12] van der Meer RW, Doornbos J, Kozerke S, Schär M, Bax JJ (2007). Metabolic imaging of myocardial triglyceride content: reproducibility of 1H MR spectroscopy with respiratory navigator gating in volunteers. Radiology.

[13] McGavock JM, Lingvay I, Zib I, Tillery T, Salas N (2007). Cardiac steatosis in diabetes mellitus: a 1H-magnetic resonance spectroscopy study. Circulation.

